# High-Titre Neutralizing Antibodies to H1N1 Influenza Virus after Mouse Immunization with Yeast Expressed H1 Antigen: A Promising Influenza Vaccine Candidate

**DOI:** 10.1155/2019/2463731

**Published:** 2019-01-08

**Authors:** Edyta Kopera, Konrad Zdanowski, Karolina Uranowska, Piotr Kosson, Violetta Sączyńska, Katarzyna Florys, Bogusław Szewczyk

**Affiliations:** ^1^Institute of Biochemistry and Biophysics, Polish Academy of Sciences, Pawinskiego 5A, 02-106 Warsaw, Poland; ^2^Institute of Chemistry, University of Natural Sciences and Humanities, 3 Maja 54, 08-110 Siedlce, Poland; ^3^Department of Recombinant Vaccines, Intercollegiate Faculty of Biotechnology, University of Gdansk and Medical University of Gdansk, Abrahama 58, 80-307 Gdansk, Poland; ^4^Mossakowski Medical Research Centre Polish Academy of Sciences, Pawinskiego 5, 02-106 Warsaw, Poland; ^5^Institute of Biotechnology and Antibiotics, Staroscinska 5, 02-516 Warsaw, Poland

## Abstract

H1N1 influenza virus is still regarded as a serious pandemic threat. The most effective method of protection against influenza virus and the way to reduce the risk of epidemic or pandemic spread is vaccination. Influenza vaccine manufactured in a traditional way, though well developed, has some drawbacks and limitations which have stimulated interest in developing alternative approaches. In this study, we demonstrate that the recombinant H1 vaccine based on the hydrophilic haemagglutinin (HA) domain and produced in the yeast system elicited high titres of serum haemagglutination-inhibiting antibodies in mice. Transmission electron microscopy showed that H1 antigen oligomerizes into functional higher molecular forms similar to rosette-like structures. Analysis of the N-linked glycans using mass spectrometry revealed that the H1 protein is glycosylated at the same sites as the native HA. The recombinant antigen was secreted into a culture medium reaching approximately 10 mg/l. These results suggest that H1 produced in *Pichia pastoris* can be considered as the vaccine candidate against H1N1 virus.

## 1. Introduction

Influenza is an infectious disease occurring around the world both in humans and animals. Influenza epidemics occur every year, causing high morbidity and mortality. Since 1918, two subtypes of haemagglutinin (HA) (H1 and H3) and two subtypes of neuraminidase (NA) (N1 and N2) have always been found in the human population [1, 2]. Vaccination is still the most effective way of protecting against the influenza infection and a way to reduce the risk of an epidemic or pandemic. Classical influenza vaccines are produced by culturing the virus in embryonated eggs and subsequently inactivating the virus after purification. However, the time required to produce the vaccine is 7-8 months, and this has always been the Achilles' heel of the traditional approach. Mutations during virus growth in the eggs have been reported to reduce the effectiveness of the influenza vaccine [3]. To overcome the egg-dependent production of influenza vaccines, several novel strategies have been provided. As the influenza virus neutralizing antibodies currently are directed primarily against the haemagglutinin, recombinant HA-based vaccines provide a promising alternative for influenza vaccine manufacture. Such a vaccine comprises a recombinant haemagglutinin obtained by genetic engineering using various expression systems [4–10].

Haemagglutinin is a homotrimeric glycoprotein, most prolifically found on the surface of the virus. It occurs in homotrimeric form. Each monomer consists of two subunits—HA1 and HA2—linked by a disulphide bond. A monomer molecule is synthesized as an inactive precursor (HA0). The protein undergoes N-linked glycosylation, and this posttranslational modification has been shown to play an important role in the proper folding, trimer stabilization, and elicitation of neutralizing antibodies [11–14].

A challenging task for the production of subunit vaccine is the development of a simple and efficient purification process for the desired antigen. The final vaccine product should contain only highly purified compound. In our study, we utilized *Pichia pastoris* cells. This expression system enables efficient secretion of the overexpressed polypeptide facilitating purification of the protein product. *Pichia pastoris* offers the possibility to produce a high level of the desired protein and is suitable for large-scale production since *Pichia* cells can easily grow in a fermenter [15–17]. Several attempts have been made to utilize the *P. pastoris* system for HA polypeptide production. The full-length HA protein of H1N1 [18, 19] and H5N2 virus [20] was expressed in *P. pastoris* as partially secreted proteins. However, the levels of expression appeared to be very low. Expression of the H5 antigen was also reported by Subathra and colleagues [21], but the protein was not exported out of the cells, which hindered its purification process.

The aim of this study was to test an H1N1pdm09 influenza virus HA produced in a yeast expression system as a potential vaccine antigen. Our previous study showed that the H5 antigen produced in the *P. pastoris* cells is capable of inducing a specific immune response in mice [8, 10] and providing full protection in chicken [9]. Ease of preparation, low cost of production, and high immunogenicity of the yeast-derived antigen prompted us to test an H1N1pdm09 influenza virus antigen.

## 2. Results

### 2.1. Purification of Yeast-Derived H1 Antigen

**Figure 1:**
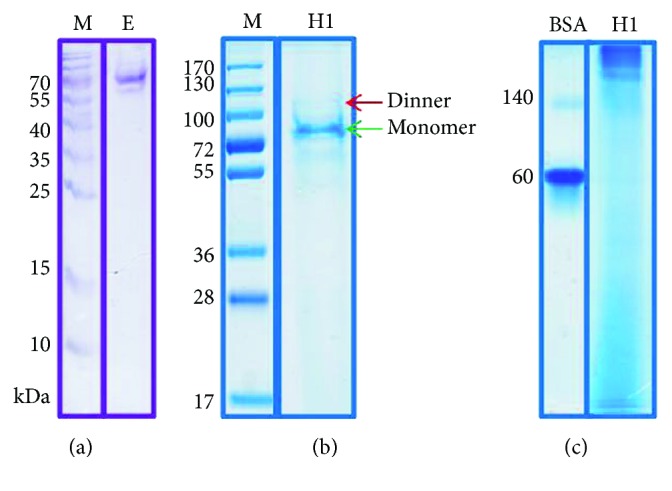
SDS-PAGE analysis of H1 antigen after IMAC purification. Protein was eluted with 250 mM imidazole. Collected fractions were analysed on 4-12% SDS-PAGE following Coomassie staining in reducing (a) and nonreducing (b) conditions. M: molecular weight marker; E: H1 protein eluted from Ni-NTA column by 250 mM imidazole. Fractions with H1 protein were pooled and lyophilized after being run on 4-7% Native PAGE (c).

Our previous results showed that the recombinant H5 protein encompassing residues from the extracellular domain adopted the correct three-dimensional structure required for oligomerization. Moreover, the H5 vaccine produced in *Pichia* cells proved to be protective for chickens challenged with a lethal dose of the highly pathogenic H5N1 virus [9]. Therefore, in this study, the transmembrane region and cytoplasmic tail of the H1 protein were also excluded. *In silico* analysis of the amino acid sequence of H1N1 haemagglutinin (A/H1N1/Gdansk/036/2009) revealed that the extracellular domain of H1 haemagglutinin comprises amino acids from 18 to 540. A DNA fragment encoding this amino acid sequence of HA (with His_6_-tag at the N-terminus) was cloned as *EcoR*I and *Sac*II insert in the pPICZ*α*A expression vector. As expected, the majority of expressed H1 represented an uncleaved HA polypeptide. No significant proteolytic processing into the HA1 and HA2 domains was detected. Coomassie blue staining after the SDS-PAGE separation of the purified H1 protein showed a high level of purity after one-step purification (Figure 1(a)). Furthermore, the purified H1 antigen typically migrates as a mixture of monomers and disulphide-linked dimers under nonreducing conditions (Figure 1(b)).

### 2.2. Characterization of H1 Antigen

**Figure 2:**
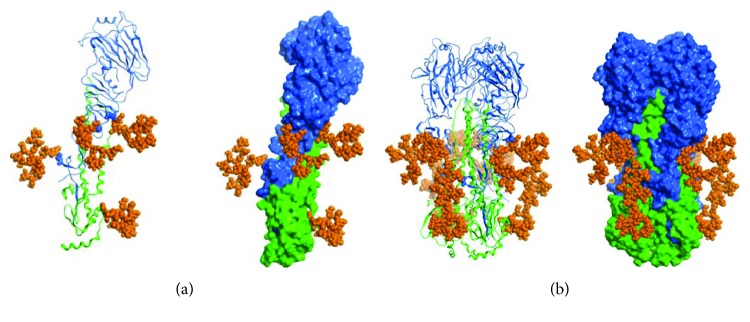
Structural representation of the molecular model of the N-glycosylated H1 monomer (a) and trimer (b). The HA1 domain is depicted with the blue ribbon, the HA2 domain is depicted with the green, and the oligosaccharides are represented by orange atomistic balls. The sugar-bonded asparagines are depicted in atomistic representations, according to their respective domain colours. The models were constructed based on the crystallographic structure of the H1 haemagglutinin (PDB ID code 3LZG, [27]).

The theoretical molecular weight of His-tagged HA lacking a C-terminal transmembrane anchor and cytoplasmic domain is 59.5 kDa. SDS-PAGE analysis showed that the H1 protein displayed a band of 70 kDa. The difference between the calculated molecular weight of H1 and the apparent molecular weight of the SDS-PAGE band indicates protein modification. Haemagglutinin is a glycosylated protein. In silico analysis of the H1 sequence using the NetNGlyc tool revealed 7 potential N-glycosylation sites (six in the HA1 domain and one in the HA2 domain). Using LC/MS-MS analysis, we examined the carbohydrate profile of the H1 antigen. Four of the seven potential N-glycosylation sites of yeast-produced recombinant H1 antigen (N39, N292, N303, and N497) were glycosylated (Table 1 and Figure 2).

**Figure 3:**
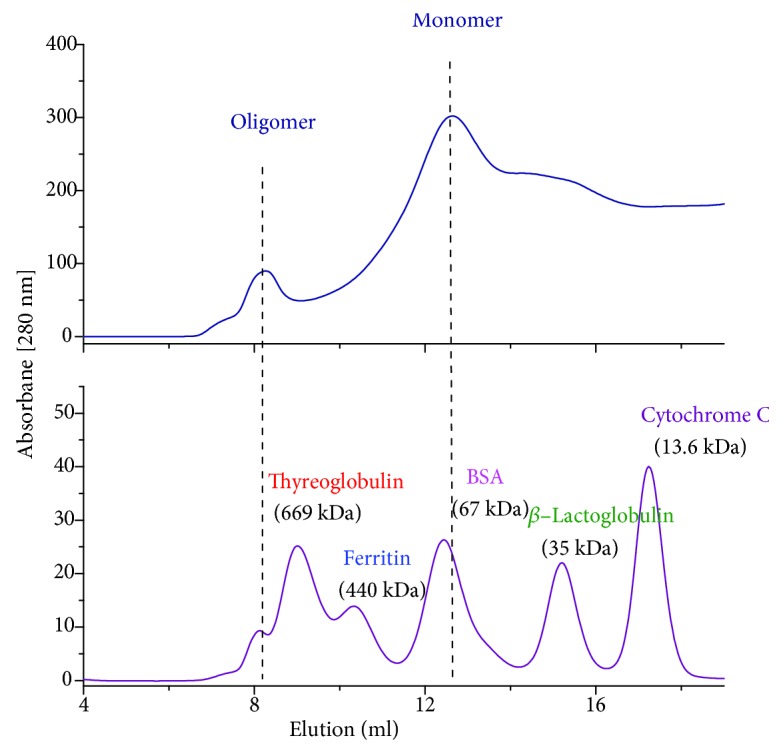
Size exclusion chromatography of the H1 antigen on Superdex 200 10/300 GL column. Chromatogram of the IMAC: elution fractions (upper plot) and molecular weight standards (lower plot). Fractions of the H1 protein were liophilised and dissolved in water, followed by injection into a Superdex 200 10/300 GL column preequilibrated with 10 mM Tris pH 7.6 with 200 mM NaCl. Absorbance at 280 nm is shown.

Electrophoresis under native conditions allowed visualization of the high molecular weight molecules in the sample eluted from the affinity column (Figure 1(c)). The recombinant antigen appeared to be a mixture of various oligomeric forms. The same elution fractions of the recombinant antigen were further analysed by size exclusion chromatography (SEC), revealing two major peaks of the H1 antigen (Figure 3). The first peak, eluted prior to a thyroglobulin molecular weight standard (~670 kDa), demonstrates that the recombinant antigen forms high molecular weight complexes. The second peak, eluted between *γ*-globulin (158 kDa) and ovalbumin (44 kDa) molecular weight standards, indicates the presence of monomers.

**Figure 4:**
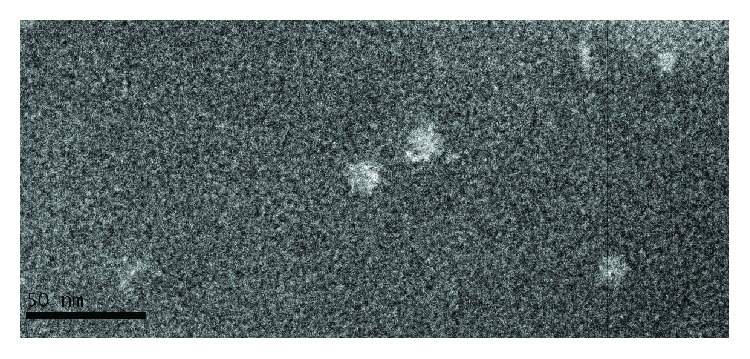
Transmission electron microscopy of the purified H1 antigen. Image was obtained at nominal 30000 magnification. The black scale bar represents 50 nm. Rosette-like structures were visualized.

Transmission electron microscopy (TEM) showed that oligomers eluted from the SEC column formed rosette-like structures (Figure 4).

**Figure 5:**
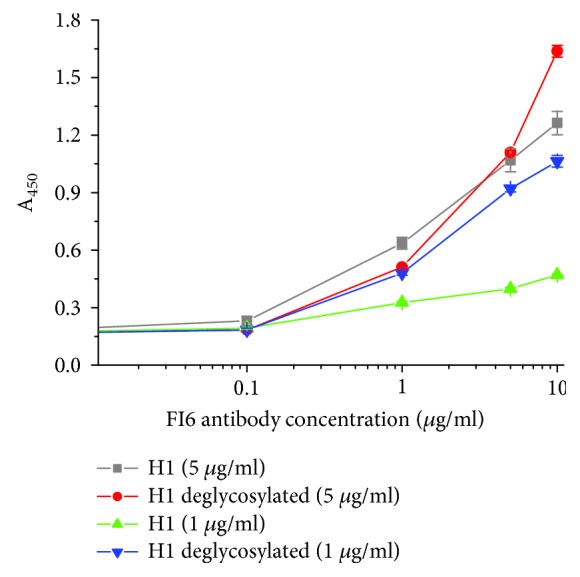
ELISA of the glycosylated and deglycosylated H1N1pdm09 recombinant protein using FI6 antibody. The H1 protein was deglycosylated with Endo H in native condition (pH 7.0). Two concentrations of the H1 protein were tested. Efficient HA-mediated haemagglutination was observed at high H1 concentration (data not shown). This is consistent with previous data, which suggested that only high HA oligomers are able to bind to multiple red blood cells to create the latticed structures that are measured in the haemagglutination assay [10].

In a modified ELISA, we examined whether H1N1pdm09 recombinant protein reacts with FI6 human neutralizing antibody. We performed a binding analysis of the glycosylated and deglycosylated H1 antigen with neutralizing antibody. The reactivity of the FI6 antibody with the glycosylated H1 antigen was observed only at a high protein concentration. Much better results were seen for the deglycosylated H1N1pdm09 recombinant protein (Figure 5).

### 2.3. Immunization of Mice with H1 Vaccine Generated High Titres of Anty-H1N1pdm09 Antibodies

**Figure 6:**
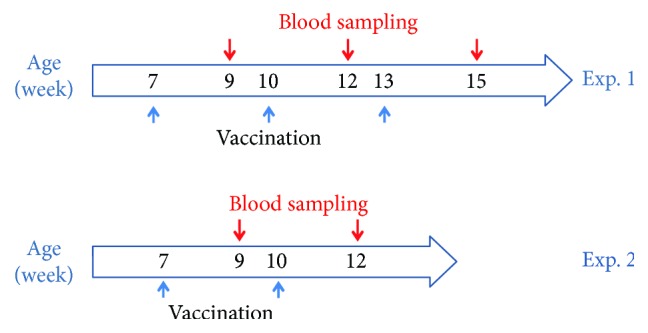
Scheme of mouse vaccination with H1 antigen. Exp. 1: mice were vaccinated subcutaneously (blue arrows) three times at three-week intervals. Blood samples were taken two weeks after each injection (red arrows). Exp. 2: mice were vaccinated intradermally twice at three-week intervals. Blood samples were taken two weeks after each injection (red arrows).

Immunogenicity studies in mice were conducted to evaluate the immunogenic potential of the H1 antigen and to determine the minimal effective dose that generates protective correlates of immunity. Two sets of immunization experiment were performed using prime/boost strategy. In the first immunization, experiment groups of mice were vaccinated subcutaneously with different doses of the H1 antigen (ranging from 1 *μ*g to 25 *μ*g) and boosted twice at three-week intervals (Figure 6, Exp. 1).

**Figure 7:**
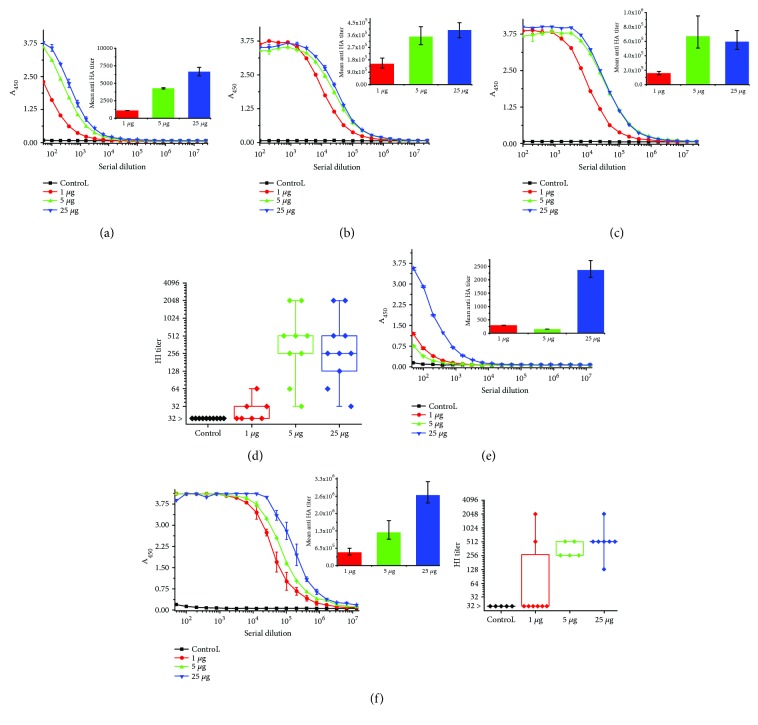
Humoural response in mice after immunization with the H1 antigen. Sera samples of mice immunized with 1, 5, and 25 *μ*g of H1 adjuvanted with Alhydrogel were pooled and the immune responses were measured two weeks after immunization (a), booster (b), and second booster (c) by indirect ELISA (nonlinear fitting plots: bars represent standard deviation; column plots: bars represent 95% confidence level) and two weeks after second booster by HI test (d). Data for individuals (raw data, ♦), mean (○), and the medians (□) are shown for each group. During the second immunization experiment, two doses of 1, 5, and 25 *μ*g of H1 adjuvanted with Sigma Adjuvant System were tested. Sera samples of mice immunized twice were measured two weeks after the first antigen injection (e) and booster (f) by indirect ELISA (bars represent 95% confidence level). Two weeks after booster, the HI test was performed with homologous H1N1pdm09 virus (f). Data for individuals (raw data, ♦), mean (○), and the medians (□) are shown for each group.

The control group received only saline plus Alhydrogel. Following immunization, the mice showed no adverse effects suggesting that the H1 vaccine was well tolerated. The results of the ELISA test demonstrated that the H1 antigen is strongly immunogenic. A single administration of the H1 vaccine at a dose as low as 1 *μ*g in a presence of aluminium hydroxide elicited specific anti-HA antibodies with an endpoint titre of 2 × 10^3^. A second antigen injection significantly boosted the antibody response in all groups, and strong dose dependence was observed. Interestingly, after the third injection, the highest HA-specific IgG titre of 6 × 10^5^ was detected in mice immunized with 5 *μ*g of the H1 vaccine (Figures 7(a)–7(c)). No increase was observed in the serum IgG response in the control mice on any of the days tested. The sera from the individual groups were pooled for testing using ELISA. For the HI assay, the sera of individual mice were tested to pinpoint the differences in protective efficacy of each of the H1 vaccine doses. The level of HI antibodies was tested after the final vaccine doses. HI antibody titres were not very high in the group immunized with 1 *μ*g, and only 40% of this group was positive. Groups of mice immunized with 5 *μ*g and 25 *μ*g of H1 vaccine were 100% positive, with HI titres as high as 1 : 2048, and no significant differences among these two groups were detected (Figure 7(d)). These results prompted us to study the protective efficacy of only one booster of the H1 vaccine. The second immunization experiment was similar to the previous one; however, a different adjuvant was used. Based on our results obtained from the immunization experiment with H5 antigen [8], the H1 vaccine formulation contained the Sigma Adjuvant System. The same doses of the H1 antigen were tested. The mice were immunized intradermally twice, at three-week intervals (Figure 6, Exp. 2). A single immunization with the H1 vaccine in the presence of the Sigma Adjuvant System elicited a rather low level of specific anti-HA antibodies, and titre < 2 × 10^3^ was detected only for the 25 *μ*g dose of the H1 antigen (Figure 7(e)). A massive increase in the immune response was detected after the booster in all three groups of immunized mice (Figure 7(f)). The HA-specific IgG titre, which was as high as 2.6 × 10^6^, was detected in mice immunized with 25 *μ*g of the H1 vaccine. No immune response to the HA antigen was detected in the control group. HI titres (determined for individual animals) were also very high. As in the first immunization experiment, most of the mice immunized with 1 *μ*g of H1 vaccine were HI negative. One hundred percent of the animals in groups immunized with 5 *μ*g or 25 *μ*g of H1 vaccine were HI positive, and no significant difference was observed in the HI titres between these two doses (Figure 7(f)). These results suggest that two doses of 5 *μ*g of the H1 vaccine might be protective against H1N1pdm09 virus infection.

## 3. Discussion

Influenza haemagglutinin is the primary target of almost all neutralizing antibodies, and it is regarded as a crucial component of current influenza vaccines. Previously, we showed that immunization with the subunit vaccine based on the extracellular region of the H5 haemagglutinin with deletion of the multibasic cleavage site elicited serum haemagglutination-inhibiting antibodies and fully protected chickens from lethal infections by the highly pathogenic H5N1 virus [9]. We also demonstrated the feasibility of producing H5N1 HA antigen in yeast [9, 10]. Therefore, testing this expression system for production of other influenza antigens, especially those HAs which are components of strains typical for humans, was successful. The biochemical and immunological characterization of purified *Pichia*-produced H1 antigen was achieved in this study.

The level of H1 protein oligomerization has been evaluated using various techniques: electrophoresis under native conditions, size exclusion chromatography, and transmission electron microscopy. Based on these results, one can conclude that most H1 antigen exists in a monomeric form; however, the higher molecular weight species are also present. Transmission electron microscopy visualized rosette-like structures of the H1 protein. We also examined whether the extracellular region of H1 protein preserves the conformational epitope. For this analysis, we used the FI6 antibody. This antibody was originally isolated from plasma cells from blood samples of donors exposed to pH1N1 [22]. FI6 targeted a distinct site on the stem region of HA and may be able to neutralize the majority of influenza A virus subtypes [23, 24]. According to reports, FI6 may be used to test the correct immunogenic conformation of HA [25]. Therefore, we used this antibody to study FI6 reactivity with recombinant H1 antigen. A relatively good reactivity of FI6 antibody with the deglycosylated H1 protein was observed suggesting that the binding site for the conformational antibody was preserved. The reactivity of the FI6 antibody with the glycosylated H1 antigen was rather poor, especially at low protein concentration. This result was consistent with the structural model of the N-glycosylated H1 protein, since all the glycosylated sites are located in its stem region. However, for both H1 protein states, low signals from FI6 might be explained by the oligomeric forms of the recombinant antigen.

Our immunization experiments showed that the H1 antigen induced a strong HI-immune response in mice. The mean anti-HA antibody titre as high as 8 × 10^5^ after the third dose of the H1 vaccine adjuvanted with Alhydrogel was detected. An even higher mean anti-HA antibody titre (6 × 10^6^) was detected after the second dose of the H1 vaccine adjuvanted with the Sigma Adjuvant System. Most anti-HA neutralizing antibodies are conformation dependent. Antibodies that are generated against haemagglutinin, especially against the receptor-binding region, have a high neutralizing potential and are able to prevent viral infection. These antibodies can be easily quantified using the HI test. A strong correlation of protection exists for serum HI titres, and this assay is commonly used for evaluation of effectivity of influenza vaccines [FDA, https://www.fda.gov/downloads/BiologicsBloodVaccines/guidanceComplianceRegulatoryInformation/Guidances/Vaccines/ucm091990.pdf]. HI tests performed by us showed that the H1 antigen induced high neutralizing antibodies titre.

The crucial issue for a vaccine to be licensed for medical use is to develop an effective process for the purification of the protein product [10]. Our work addresses both vaccine production steps: the expression and the purification. In this study, we investigated the use of the *Pichia* cells to produce a soluble H1N1 HA antigen. The *P. pastoris* expression system has been commonly utilized as a platform to produce various proteins significant to the medical industry, including vaccine antigens (Shanvac™, Elovac™, and Gavac™) [10]. We proved that the *Pichia*-based production process is also effective for the H1 antigen. The manufacturing method is simple and inexpensive compared to the existing solutions. A good efficiency of up to 10 mg of highly purified protein from 1 litre of culture medium (400 doses) for the H1 antigen was obtained. The efficiency of the H1 vaccine antigen production presumably could be easily scaled up in a bioreactor.

## 4. Materials and Methods

### 4.1. Production and Purification of H1 Antigen

Plasmid pPICZ*α*A (Invitrogen) and plasmid pJET/H1 carrying H1N1 virus haemagglutinin gene (A/H1N1/Gdansk/036/2009) have been used for the construction of the expression vector pPICZ*α*A/H1. The plasmid contains an *α* factor sequence, which ensures the secretion of recombinant protein to the medium. cDNA of 1569 bp, which codes the hydrophilic domain (18-540 aa) of the H1N1 virus haemagglutinin (A/H1N1/Gdansk/036/2009), was amplified in a PCR reaction. The sequence encoding the His-Tag was added to the cDNA sequence by forward primer. The PCR product was cloned into the pPICZ*α*A expression vector under the control of the AOX promoter. *Pichia pastoris* cells (KM 71 strain, his4, aox1::ARG4, arg4) (Invitrogen) were transformed with recombinant plasmids by electroporation. Integration of the haemagglutinin gene into the *P. pastoris* genome was confirmed by PCR. KM 71 transformant selection was performed using a medium containing Zeocin (multiple passaging). The presence of recombinant protein, in the medium and in the cells (control), was detected by SDS-PAGE and Western blotting. Recombinant haemagglutinin was purified from culture medium by affinity chromatography, using Ni-NTA Agarose resin. Protein binding to the Ni-NTA Agarose was carried out in phosphate buffer solution (PBS) with 450 mM NaCl. The HA protein was eluted from the column with 250 mM imidazole in PBS pH 7.4. The protein was then dialyzed against PBS pH 7.4. After dialysis, the protein was lyophilized and stored at a temperature of −20°C.

### 4.2. Enzymatic and MS/MS Analysis of Recombinant HA Glycosylation

5 *μ*g of H1 protein was incubated with 750 U of endoglycosidase H (New England BioLabs) at 37°C for 18 h. The reaction samples were analysed by Western blotting and SDS-PAGE after. A band corresponding to the deglycosylated HA protein was cut from the gel for MS/MS analysis. The protein was digested by trypsin. The mixture of peptides (without reduction or alkylation) was applied in an RP-18 precolumn (Waters nanoAcquity 20 mm × 180 *μ*m) with water and 0.1% trifluoroacetic acid and then into an HPLC RP-18 column (Waters nanoAcquity UPLC column 250 mm × 75 *μ*m), using a line gradient of acetonitrile (0-50% in 30 minutes) with 0.1% formic acid. The elution fraction from the HPLC column was directed to the MS ionization chamber (LTQ-FTICR, Thermo Electron). The Protein Bank Data PDB (National Center of Biotechnology Information NCBI no. Version 20080624) and Mascot software (http://www.matrixscience.com) were used for the analysis of the resulting mass spectra.

### 4.3. Size Exclusion Chromatography

The H1 antigen was analysed according to a procedure previously published [9]. Shortly, a Superdex 200 10/300 GL column (GE Healthcare, UK) was preequilibrated with 10 mM Tris pH 7.6 with 200 mM NaCl, and the protein was injected with the equilibration buffer. Elution of the H1 antigen was monitored at 280 nm. Molecular weight standards (Bio-Rad, USA) were used to calibrate the column and to identify the molecular weights of the proteins present in the samples.

### 4.4. Transmission Electron Microscopy

TEM analysis was performed in collaboration with Electron Microscopy platform of the Integrated Structural Biology of Grenoble. A procedure previously published was applied [9]. Shortly, 100 *μ*g of the H1 protein sample was applied to the clean side of the carbon on mica and negatively stained with 2% (w/v) sodium silico tungstate. A grid was then placed on top of the carbon film which was subsequently air-dried. Images were taken under low-dose conditions (less than 20 e−/A2) with a T12 FEI electron microscope at 120 kV using an ORIUS SC1000 camera (Gatan, Inc., Pleasanton, CA).

### 4.5. Immunization

Seven-week-old, pathogen-free, female BALB/c mice were used for vaccination. The animals were housed in a temperature-controlled environment at 24°C with 12 h day-night cycles and received food and water *ad libitum*. Immunization experiments were conducted in the animal house of the Institute of Experimental Medicine PAS (Warsaw) under control of the Bioethics Committee (Permission no. 11/2012). The experimental groups in the first trial consisted of 10 mice. The control group of 10 mice was administered adjuvant only. The recombinant antigen suspended in saline solution supplemented with Alhydrogel (aluminium hydroxide) was administered subcutaneously (sc) into the neck skin-fold. The antigen was administered at three doses: 1, 5, and 25 *μ*g. Sera samples were taken from all groups one week before the application of the first dose. There were three injections (first application of antigen and/or adjuvant + two booster shots) at an interval of three weeks between each dose in order to monitor immunological response. Blood samples were taken two weeks after each injection to determine the level of antibodies. Sera were stored at −20°C. The experimental groups in the second immunization experiment consisted of 8 7-week-old BALB/c mice. Five mice were in the control group. The antigen was administered at three doses, the same ones as described above. For immunization, 25 *μ*g of the Sigma Adjuvant System containing 0.5 mg monophosphoryl Lipid A (detoxified endotoxin) from *Salmonella minnesota* and 0.5 mg synthetic Trehalose Dicorynomycolate in 2% oil (squalene)-Tween® 80-water was used. The booster injection was adjuvanted with 25 *μ*g monophosphoryl lipid A and 25 *μ*g muramyl dipeptide (Sigma-Aldrich). The control group was administered with only the specific adjuvant. Administration of 100 *μ*l of vaccine was by intradermal injection (i.d.) in the walking pad of the hind paw. As in the first experiment, blood samples were taken two weeks after each injection in order to determine the level of antibodies. Sera were stored at −20°C.

### 4.6. ELISA

Collected sera were assayed for antibodies against H1 HA by an ELISA method, using MaxiSorp plates (Nunc, Denmark) coated with purified HA (coating concentration 1.6 *μ*g/ml). Sera samples from mice immunized with the H1 protein were tested in parallel with sera from sham-immunized mice (negative controls). A procedure previously published was applied [10]. Sera samples, taken from individual mice at each time point of the experiment, were pooled in groups, serially diluted in 2% BSA/PBS and applied onto the plates (overnight, 2-8°C) and blocked with 2% BSA/PBS (1.5 h, 37°C). The tested samples were then incubated overnight at 2–8°C together with blanks (sample diluent). Bound antibodies were subsequently detected with goat-generated and horseradish peroxidase- (HRP-) labelled antibodies against mouse IgG (*γ*-chain specific) at 1 : 1000 dilution in 2% BSA/PBS (1 h, 37°C). TMB was used as a HRP substrate. After incubation for 30 min at room temperature, the reaction was stopped by the addition of 0.5 M sulfuric acid. The absorbance was measured at 450 nm with a microplate reader (Synergy 2; BioTek Instruments, USA). The endpoint titre was defined as the highest dilution producing an A_450_ value 4-fold higher than the mean A_450_ value of the control group.

In an enzyme-linked immunosorbent assay (ELISA), 50 *μ*l of FI6 antibody at the concentrations of 10 *μ*g/ml-0.01 *μ*g/ml was coated on 96-well ELISA MediSorp plates (Nunc, Denmark) and allowed to bind overnight at 4°C. Plates were then washed four times with washing buffer (PBS, 0.1% Tween, pH 7.6) and blocked with 2% BSA/PBS (blocking buffer) at 37°C for 1.5 h. After the plates had been washed two times with washing buffer, 50 *μ*l of glycosylated or deglycosylated H1 antigen (5 *μ*g/ml or 1 *μ*g/ml) in PBS was then added to the plates and incubated at room temperature for 1.5 h. Plates were again washed four times with washing buffer. Bound antigens were detected with horseradish peroxidase- (HRP-) labelled antibodies against His-Tag at 1 : 5000 dilution in 2% BSA/PBS (37°C, 1 h). After incubation for 30 min at room temperature with the TMB, 0.5 M sulfuric acid was added to stop the reaction. The absorbance was measured at 450 nm with a microplate reader (Synergy 2; BioTek Instruments, USA).

### 4.7. Haemagglutination Inhibition Test

Sera samples were heat inactivated at 56°C for 30 min and then were pretreated with kaolin to avoid a false-positive reaction in the HI test [26]. The pretreated sera samples (25 *μ*l of sera in serial twofold dilutions) were incubated for 25 min in a titration plate with 4 HA units of the inactivated antigen [10]. Next, the suspension of 1% hen erythrocytes was added and incubated for 30 min. The HI titre was determined as the reciprocal of the highest dilution in which haemagglutination is inhibited. Samples were assigned as positive when their titre was ≥16. Sera samples from the immunization experiments (trials 1 and 2) were tested using the homologous strain H1N1.

## Figures and Tables

**Table 1:** N-glycosylated peptides from the H1 protein confirmed by LC/MS/MS analysis. *N*-linked glycosylation sites are underlined.

Residue	Region	Amino acid sequence
N39	HA1	NVTVTHSVNILEDK
N292	HA1	NAGSGIIISDTPVHDCNTTCQTPK
N303	HA1	GAINTSLPFQNIHPITIGK
N497	HA2	NGTYDYPK

## Data Availability

The models were constructed based on the crystallographic data deposited in PDB (ID code 3LZG).

## Abbreviations

HA: Haemagglutinin
AOX: Alcohol oxidase
Endo H: Endoglycosidase H
IV: Influenza virus
PAGE: Polyacrylamide gel electrophoresis
SEC: Size exclusion chromatography
TEM: Transmission electron microscopy
HI: Haemagglutination inhibition
IMAC: Immobilized metal ion affinity chromatography.
